# Ghrelin Inhibits Post-Operative Adhesions via Blockage of the TGF-β Signaling Pathway

**DOI:** 10.1371/journal.pone.0153968

**Published:** 2016-04-15

**Authors:** Enrica Bianchi, Kim Boekelheide, Mark Sigman, Dolores J. Lamb, Susan J. Hall, Kathleen Hwang

**Affiliations:** 1 Division of Urology, Brown University, Providence, Rhode Island, United States of America; 2 Department of Pathology and Laboratory Medicine, Brown University, Providence, Rhode Island, United States of America; 3 Center for Reproductive Medicine, Scott Department of Urology, Department of Molecular and Cellular Biology Baylor College of Medicine, Houston, Texas, United States of America; National Cancer Institute, UNITED STATES

## Abstract

Post-operative adhesions are a critical problem in pelvic and abdominal surgery despite a multitude of studies dedicated to finding modalities to prevent their occurrence. Ghrelin administration promotes an anti-fibrotic response in a surgical mouse model of adhesion-induction, but the mechanisms mediating this effect have not been established. In the current study, the molecular mechanisms that underlie the anti-adhesion effect of ghrelin were investigated. Post-surgical adhesions were experimentally created in C57BL/6 wild-type mice via a combination of ischemic peritoneal buttons and cecal multiple abrasions. Ghrelin or saline intraperitoneal injections were given twice daily from two days before surgery to selected time points post-surgically to assess the phenotypic and molecular effects of treatment (1 day (n = 20), 4 days (n = 20) and 20 days (n = 40) after surgery). Endpoints included the scoring of adhesions and gene and protein expression analysis of pro-fibrogenic factors conducted on peritoneal ischemic tissue by quantitative PCR and Western blot. Ghrelin administration significantly reduced post-surgical adhesions and down-regulated pro-inflammatory gene and protein expression, including Tgfb3 and Tgfbr2. The up-regulation of inhibitory proteins Smad6 and Smad7 confirmed the ghrelin-induced blockage of TGF-β signaling. Ghrelin is a candidate therapeutic drug for post-operative adhesion prevention, inhibiting inflammatory responses via blockage of the TGF-β signaling pathway at the onset of surgery before the occurrence of the granulation-remodeling phase.

## Introduction

After pelvic or abdominal surgery, post-surgical adhesions are formed when abnormal fibrous connective tissue is produced by extracellular matrix secretion, fibrinolysis and neo-angiogenesis. Several pathways are involved in adhesiogenesis, many of which are also involved in normal wound healing [1]. Despite the diverse strategies developed to minimize and prevent post-operative adhesions in pelvic and abdominal surgery, adhesions remain a frequent complication [2].

The pathogenesis of post-operative adhesion formation is similar after almost all types of surgery. Surgical trauma evokes an inflammatory response, promoting pro-coagulatory and anti-fibrinolytic reactions causing an increase in fibrin formation. Peritoneal inflammation is a crucial factor in determining the degree of imbalance between fibrin formation and degradation that causes adhesion formation [1]. The pathogenesis of post-operative adhesions is characterized by two prominent steps: the initial inflammatory response, in which immune cells and cytokines play crucial roles, and the granulation-remodeling phase [3].

Various signals and molecular mediators are involved in post-surgical adhesion formation. Peritoneal surgical injury initiates inflammation with fibrinous exudate and fibrin formation activated from the coagulation cascade pathway [4]. After surgery, the balance between coagulation and fibrinolysis is in favor of the coagulation system, creating a fibrin matrix. During the granulation phase, fibroblasts migrate into this fibrin matrix and differentiate into myofibroblasts causing deposition of extracellular matrix components (ECM). At this point the extracellular matrix can be completely dissolved by matrix metalloproteinases (MMPs), leading to normal wound healing or if this process is prevented by inhibitors of MMPs, peritoneal adhesions may occur [5].

Pro-inflammatory cytokines, especially transforming growth factor β (TGF-β), which is activated at the site of peritoneal injury, play an important role in regulating coagulation as well as fibrin formation, influencing the development of adhesions [6]. The TGF-β/Smads signal transduction pathway acts as an important bridge between the inflammatory response and fibrosis [7]. TGF-β stimulates fibroblast cell activation and extracellular matrix synthesis through its interaction with TGF-β receptors and activation of Smad2/3. Activation of Smad2/3 via phosphorylation induces their association with Smad4 and subsequent translocation into the nuclei, where these factors control the transcription of TGF-β–responsive genes [8]. Recruitment of inflammatory cells and expression of pro-inflammatory mediators contribute to the progression of fibrosis. When TGF-β is produced by infiltrating immune, inflammatory and mesenchymal cells, it signals transcriptional activation of pro-fibrotic genes, via the TGF-β/Smads signaling pathway or through alternative pathways such as the p38 MAPK signaling and RAS/ERK MAPK signaling pathways [9, 10]. The concentrations of INF-γ and Il17 in the supernatant fluid are maximal at 6–12 hours after surgery, whereas TGF-β1 exhibits two-post-operative peaks of secretion at 2 hours and 3–4 days [11]. Intraperitoneal injection of high doses of TGF-β3, classified as a ‘‘motogenic” factor [12], increased adhesion formation after injury of the peritoneum with enhanced collagen deposition and fibroblastic proliferation [13].

Ghrelin [14, 15], a 28-amino acid gastric peptide first isolated from the rat stomach [14], which interacts with the growth hormone secretagogue receptor 1a (GHSR1a) [16], can display anti-inflammatory [17–20] and anti-fibrotic effects [21–23]. Ghrelin circulates in two forms, ghrelin (acylated) and desacyl ghrelin [14]. Acylated ghrelin has been shown to be able to bind and activate GHSR1a due to octanoylation mediated by ghrelin O-acyltransferase [14]. Desacyl ghrelin, lacking of the post-translational modification of acylation for GHSR binding, has shown to have an effect on the cardiovascular system and metabolism of glucose and lipids [24].

Previous studies from our group showed that intraperitoneal administration of exogenous acylated ghrelin minimizes post-operative intra-abdominal adhesion formation, but the mechanism by which ghrelin impacts adhesions was not investigated [25]. A new surgical mouse model of induction of adhesions in C57BL/6 mice that is easily reproducible and effective at producing consistent adhesions for analysis was developed and characterized. This model, which showed consistency in intra-abdominal adhesion formation between cecum and peritoneal ischemic buttons, provides an excellent approach to define the ability of acylated ghrelin to prevent adhesion formation in a GHSR1a receptor-dependent manner. The involvement of GHSR1a receptor in the ghrelin-induced anti-adhesion effect was supported by using GHSR-null mice [25]. The present study evaluates the molecular mechanisms by which ghrelin impacts wound healing and peritoneal adhesions by analyzing the expression of pro-fibrogenic factors at the mRNA and protein levels at various post-operative time-points. This study indicates that the inhibition of post-operative adhesions by intraperitoneal injections of ghrelin is mediated via blockage of TGF-β pathway at the onset of surgery before the occurrence of the granulation-remodeling phase.

## Materials and Methods

### Animals

Male C57BL/6 mice (n = 80) were purchased from Charles River Laboratories (Wilmington, MA). All mice, 50–55 days old, were housed under standard conditions (room temperature 25–28°C, humidity 30–70% and 12 hr dark-light cycle) in Brown University Animal Care Facility. The mice were fed a standard rodent chow (Purina Rodent Chow 5001, Farmer’s Exchange, Framingham, MA) and *ad libitum* access to filtered tap water. The study was approved by the Brown University Institutional Animal Care and Use Committee and conducted in accordance with The Guide for the Care and Use of Laboratory Animals of the National Institute of Health. All surgery was performed under anesthesia, and all efforts were made to minimize suffering.

### Chemicals

Rat lyophilized acylated ghrelin was obtained from Tocris Bioscience (Bristol, UK).

### Experimental Design

Post-surgical intraperitoneal adhesions were induced as previously described [25]. In brief, mice underwent a laparotomy to create a combination adhesion model induced by ischemic peritoneal buttons and multiple cecal abrasions. Mice were randomly divided into two treatment groups: ghrelin group (0.16 mg/kg) and control group (saline). Mice received intraperitoneal injections of saline (0.1 ml) or 0.16 mg/kg of ghrelin diluted in 0.1 ml of saline, twice daily from 2 days pre-surgery to 1 day (n = 20), 4 days (n = 20) and 20 days (n = 40) post-surgery. Mice were euthanized by isoflurane overdose and cervical dislocation.

### Adhesion formation score

The mice were euthanized at different time-points after surgery, 1 day (ghrelin-treated mice n = 10, saline-treated mice n = 10), 4 days (ghrelin-treated mice n = 10, saline-treated mice n = 10) and 20 days (ghrelin-treated mice n = 20, saline-treated mice n = 20). Post-mortem, the adhesions were scored by a blinded surgeon according to the scoring system described by Mazuji MK et al, 1964 [26]: grade 0 = no adhesions; grade 1 = scattered filmy adhesions; grade 2 = moderately dense, scattered adhesions; grade 3 = dense, continuous adhesions; grade 4 = very dense homogenous adhesions.

### RNA and protein extraction

The two peritoneal ischemic buttons were harvested at 1 day (ghrelin-treated mice n = 10, saline-treated mice n = 10), 4 days (ghrelin-treated mice n = 10, saline-treated mice n = 10) and 20 days (ghrelin-treated mice n = 20, saline-treated mice n = 20) post-surgery. Both the ischemic buttons were frozen in liquid nitrogen. RNA was isolated from one peritoneal ischemic button using the RNeasy Micro Kit (Qiagen, Valencia, CA) with the additional recommended DNase step provided in the kit. The total RNA was reverse transcribed to cDNA using the RT^2^ first strand kit (Qiagen, Valencia, CA). The RNA were assessed based on the RIN values generated from RNA nano kit and 2100 bioanalyzer system (Agilent technologies, Santa Clara, CA). Protein lysates were prepared by homogenizing the second peritoneal button with an electric homogenizer in radioimmunoprecipitation assay buffer (RIPA buffer: 50 mM Tris-HCl (pH 8), 150 mM NaCl, 0.1% Triton X-100, 0.5% sodium deoxycholate, 0.1% SDS) with 1% phenylmethylsulfonyl fluoride (PMSF) and 1% protease and phosphatase inhibitor cocktail (Thermo Scientific, Rockford, IL). After the samples were centrifuged for 20 minutes at 12,000 rpm, supernatants were aspirated and placed in new tubes. The protein concentration was determined for each sample using Protein Assay kit (Bio-Rad, CA).

### Real-time PCR array analysis

A custom SABiosciences PCR array (Qiagen, Valencia, CA) was created incorporating 40 genes generated from a pilot study using a Mouse Fibrosis PCR Array. Each PCR plate contained an internal positive control to detect synthetic DNA (PPC) and two negative controls to detect genomic DNA contamination, a genomic DNA control (GDC) and a no reverse transcriptase control (NRT). Five housekeeping genes were chosen *(Actb*, *B2m*, *Gapdh*, *Gusb and Hsp90ab1)*. Samples were prepared according to manufacturer’s instructions, and each group was divided among the plates ensuring that each plate contained control and treated samples. Samples were placed onto 384-well plates using an epMotion® 5075 automated pipetting system (Eppendorf, Hauppauge, NY) and run on a ViiA 7 RT-PCR System (Life Technologies, Grand Island, NY) using manufacturer recommended cycling conditions. Threshold cycle (Ct) values were calculated from the amplification plot. The target gene Ct values were normalized to the geometric mean of all stable housekeeping genes through the ViiA^tm^ software AB (Applied Biosystems, Life Technologies) and analyzed using the comparative Ct method (ΔΔCt method).

### Real-time qPCR Primer Assay

Real-Time PCR was performed using primers specific for Tgfb3, Tgfbr2, Il4, tPA, Mapk14, Icam-1 and Vcam-1 from SA Biosciences (Qiagen, Valencia, CA). RT-PCR reactions were performed according to manufacturer’s instructions using RT2 SYBR Green (SA Biosciences). All assays including no template controls were done in triplicate. One stable housekeeping gene was used as internal control *(B2m)*. Samples were placed onto 96-well plates using an epMotion® 5075 automated pipetting system (Eppendorf, Hauppauge, NY) and run on a ViiA 7 Real-Time PCR System (Life Technologies, Grand Island, NY) using manufacturer recommended cycling conditions. Data were analyzed using comparative Ct method (ΔΔCt method).

### Western blot

The samples were denatured and reduced with a loading buffer containing 4% anionic denaturing detergent sodium dodecyl sulfate (SDS), 20% glycerol, 0.004% bromophenol blue, 10% β-mercaptoethanol and 0.125 M Tris-HCl (pH 6.8) and boiled at 95°C for 10 minutes. Proteins were separated by SDS-PAGE and electrotransferred to ImmunoBlot PVDF membrane (Bio-Rad, Hercules, CA). PVDF membranes were blocked with 5% non-fat milk for 1 hour at room temperature. The following primary antibodies were used: anti-TGFβ3 (Rabbit polyclonal 1:200, Abcam ab15537, Cambridge MA), anti-TGFβRII (Rabbit polyclonal 1:500, Abcam ab61213, Cambridge MA), anti-IL4 (Rabbit polyclonal 1:500, Abcam ab9728, Cambridge MA), anti-phospho-p38 MAPK (Rabbit polyclonal 1:1000, Cell Signaling Technology #9211, Danvers, MA), anti-p38 MAPK (Rabbit polyclonal 1:1000, Cell Signaling Technology #9212, Danvers, MA), anti-ICAM-1 (goat polyclonal 1:200, Santa Cruz Biotechnology sc-1511 Dallas, TX), anti-VCAM-1 (rabbit polyclonal 1:1000, Cell Signaling Technology #13662, Danvers, MA), anti-phospho-SMAD2 (Rabbit mAb 1:1000, Cell Signaling Technology #3108, Danvers, MA), anti-phospho-SMAD3 (Rabbit mAb 1:1000, Cell Signaling Technology #9529, Danvers, MA), anti-SMAD2/SMAD3 (Rabbit mAb 1:1000, Cell Signaling Technology #8685, Danvers, MA) and anti-γ-tubulin (Mouse monoclonal 1:2000, Sigma T6557, St. Louis, MO). After washing the membranes with 0.01% TBS buffer, the membranes were incubated for 1 hour at room temperature with the following secondary antibodies: HRP-conjugated anti-rabbit (#7076S Cell Signaling Technology, Boston, MA), HRP-conjugated anti-mouse (#7074S Cell Signaling Technology, Boston, MA) and HRP-conjugated anti-goat (sc-2922 Santa Cruz Biotechnology, Dallas, TX). Band intensities were quantified by densitometric analysis using Image-J software and normalized for loading. The anti-γ-tubulin antibody was used as an internal control.

### Statistical analysis

All data are presented as mean ± standard error of the mean (SEM). Statistical analysis was performed using GraphPad Prism software. Student’s t-test was used to determine statistical difference between control and ghrelin-treated groups. Values were considered to be significant at p-value < 0.05. The time course analysis between ghrelin and saline-treated mouse groups was conducted using a two-way analysis of variance (ANOVA) followed by individual comparisons using Fisher’s LSD test. Differentially expressed genes (p <0.05) were uploaded into QIAGEN’s Ingenuity Pathway Analysis software (IPA, QIAGEN Redwood City) to map genes onto pathways and identify upstream and downstream interactions.

## Results

### Ghrelin administration increased mouse body weight after surgery

**Fig 1:**
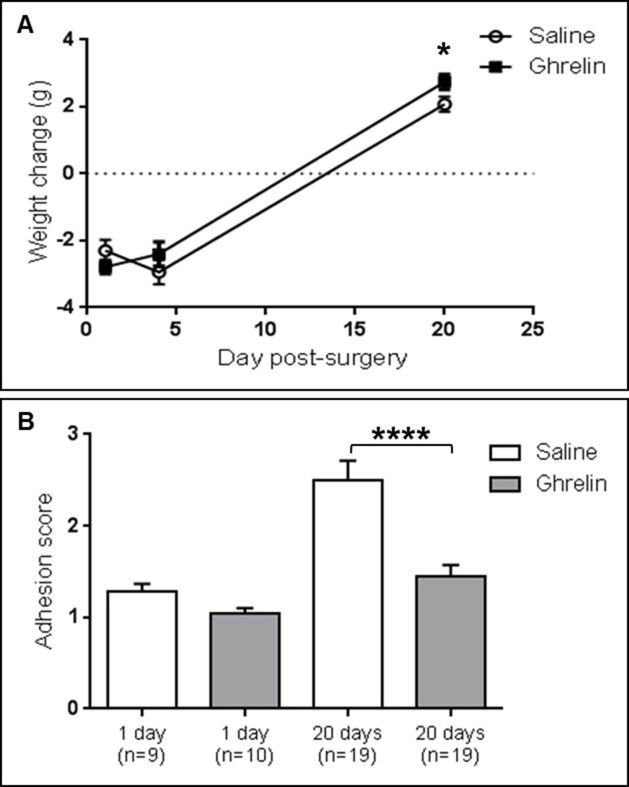
Ghrelin increased body weight gain and reduced adhesion formation at 20 days post-surgery. (A) The body weight gain in the ghrelin-treated group at 20 days post-surgery was significantly higher than in the saline-treated group. No differences were detected in the body weight gain between ghrelin and saline-treated animals at 1 day and 4 days post-surgery. (B) Ghrelin administration significantly reduces post-operative adhesion formation at 20 days post-surgery. No significant difference in adhesion score was detected at 1 day post-surgery between saline and ghrelin-treated mice. Data are analyzed by two-way ANOVA multiple comparisons Fisher’s LSD test and expressed as mean ± SEM (**** p < 0.0001, * p < 0.05).

Ghrelin administered from preoperative day 2 to 20 days after surgery significantly (p<0.05) increased the body weight in mice compared to controls (2.742 ± 0.228 g versus 2.079 ± 0.229 g, p<0.05). The ghrelin treatment-related increase of body weight in mice at 20 days post-surgery was 25% compared to controls. At 1 and 4 days after surgery mice lost weight due to decreased food intake after surgery. No significant differences in body weight gain were detected in mice treated with ghrelin compared to the saline group at 1 day (-2.790 ± 0.197 g versus -2.300 ± 0.323 g, N.S.) or 4 days (-2.400 ± 0.362 g versus -2.940 ± 0.362 g, N.S.) after surgery (Fig 1A).

### Adhesion formation was altered by ghrelin administration

Adhesion formation was measured by a blinded surgeon using a scoring system described by Mazuji MK et al, 1964 [26]. Ghrelin administered intraperitoneally twice daily from 2 preoperative days to 20 days post-surgery, significantly (p<0.0001) decreased intra-abdominal adhesions by 42.11% compared to the controls [25]. At 20 days post-surgery in the control group the adhesions were fully formed, vastly different from the saline-treated group at 1 day post-surgery. No significant differences in adhesions were detected at 1 day post-surgery between the ghrelin and saline-treated group. The adhesion scores (mean ± SEM) at 1 day and 20 days after surgery were 1.278 ± 0.088 versus 1.050 ± 0.050 (N.S.) and 2.500 ± 0.213 versus 1.447 ± 0.126 (p<0.0001), respectively (Fig 1B).

### Quantitative gene expression analysis showed altered mRNA levels in ghrelin-treated ischemic buttons

To evaluate the ghrelin-induced anti-fibrotic effect in our mouse surgical adhesion model, gene expression analysis was performed to analyze a panel of 40 genes that are involved in adhesiogenesis. Gene expression profiling was conducted on peritoneal ischemic tissue isolated from mice treated with ghrelin or saline at three different time-points: 1 day, 4 days, and 20 days post-surgery.

**Fig 2:**
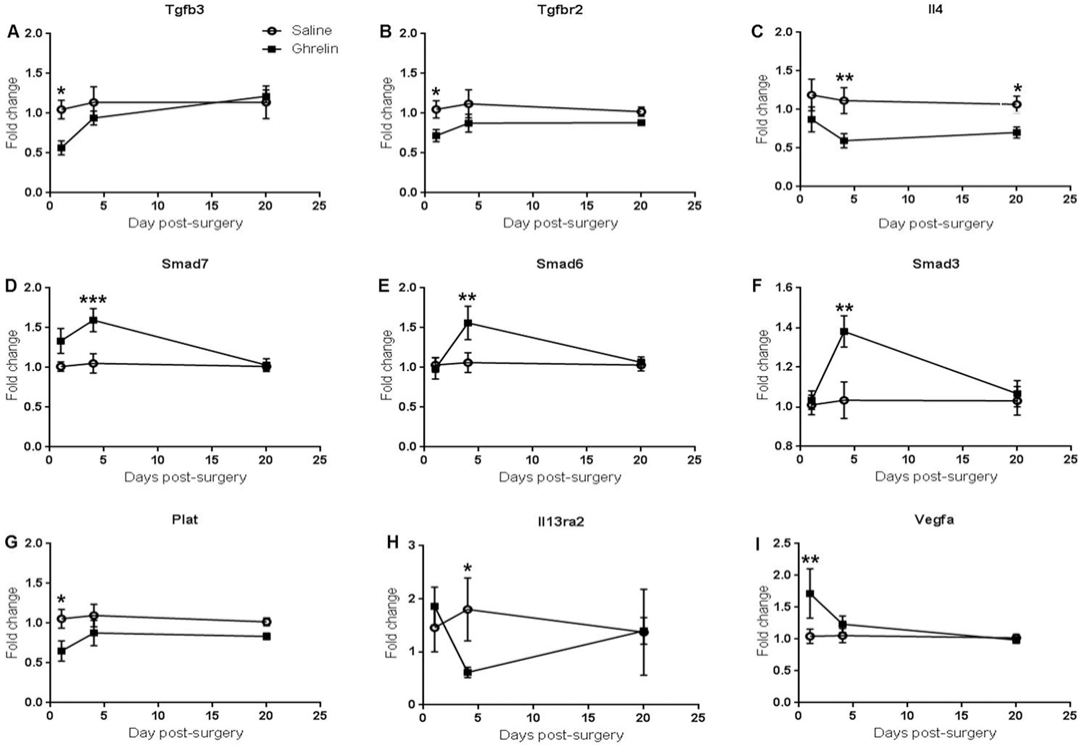
Ghrelin treatment induced time-course gene changes in peritoneal ischemic tissue. Time-course analysis of changes in expression of selected pro-fibrotic genes in ischemic buttons from ghrelin and saline-treated mice. (A) Tgfb3, (B) Tgfr2, (C) Il4, (D) Smad7, (E) Smad6, (F) Smad3, (G) Plat, (H) Il13ra2 and (I) Vegfa. Data are presented as fold change and analyzed by two-way ANOVA multiple comparisons Fisher’s LSD test (*** p <0.001, ** p < 0.01, * p < 0.05).

**Fig 3:**
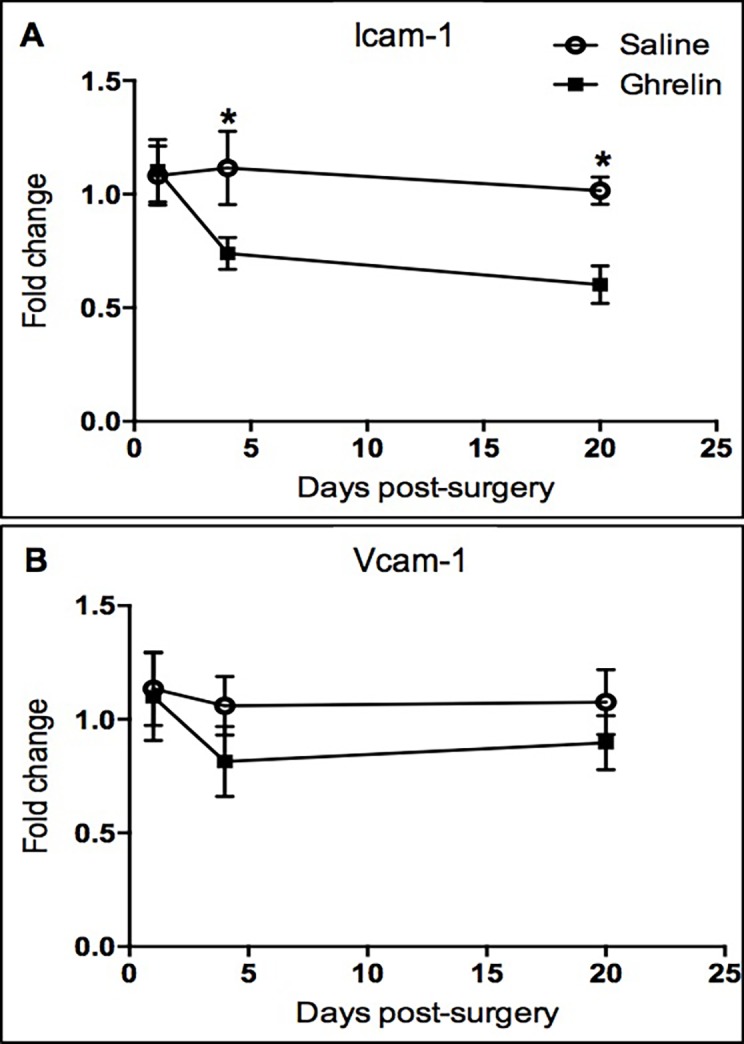
Ghrelin treatment reduced Icam-1 mRNA levels at 4 days post-surgery. Gene expression of Icam-1 (A) and Vcam-1 (B) in ghrelin- and saline-treated ischemic buttons at 1, 4 and 20 days after surgery. Data analyzed by two-way ANOVA multiple comparisons Fisher’s LSD test (* p < 0.05).

**Fig 4:**
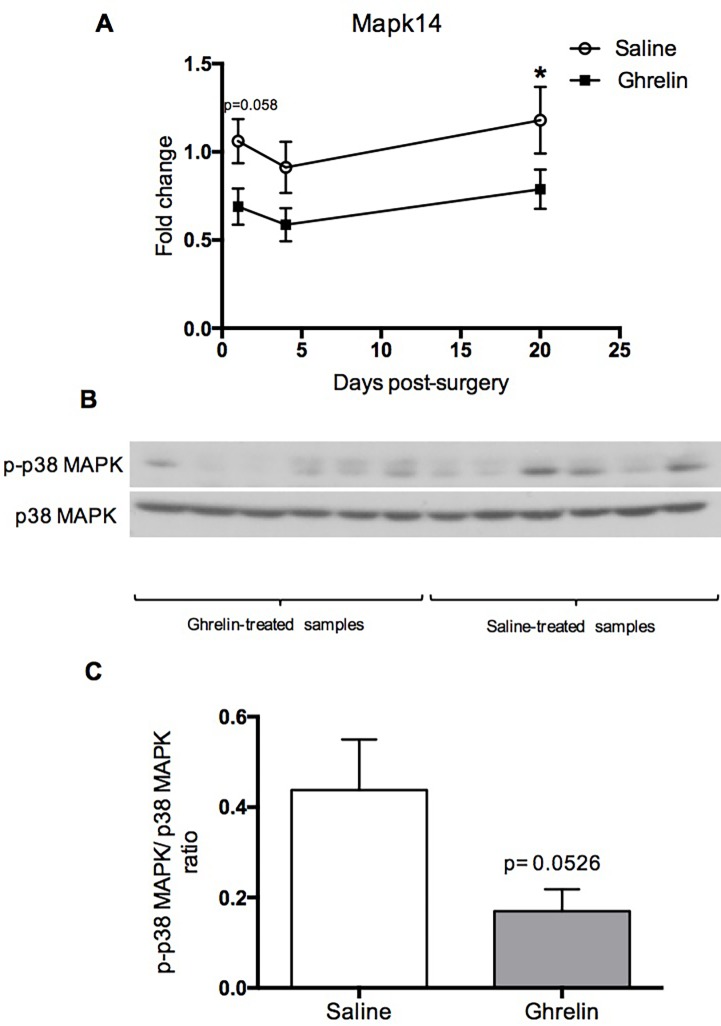
Ghrelin treatment attenuated ischemia-induced p38 phosphorylation and activation. (A) Gene expression of Mapk14 (p38α) in ghrelin- and saline-treated ischemic buttons at 1, 4 and 20 days post-surgery. Data analyzed by two-way ANOVA multiple comparisons Fisher’s LSD test (* p < 0.05). (B) Peritoneal ischemic buttons from mice treated with ghrelin or saline were subjected to Western blot analysis using antibodies against phospho-p38 MAPK and total p38 MAPK, respectively. (C) The phospho-p38/total p38 protein level ratio was reduced in ghrelin-treated samples (n = 6) compared to the controls (n = 6) at 4 days post-surgery. Data are expressed as mean ± SEM and analyzed by Student’s t-test.

The time course analysis was conducted using fold change data from qPCR at 1, 4 and 20 days post-surgery. Two-way ANOVA for individual comparisons using Fisher’s LSD test showed that ghrelin-treatment significantly decreased transforming growth factor beta 3 (Tgfb3, p = 0.0161), transforming growth factor beta receptor 2 (Tgfbr2, p = 0.0222) and tissue plasminogen activator (Plat, p = 0.0125) mRNA levels at 1 day post-surgery compared to the saline-treated samples (Fig 2A, 2B and 2G). In contrast, vascular endothelial growth factor (Vegfa, p = 0.0081) in ghrelin-treated samples was up-regulated at 1 day post-surgery compared to the controls (Fig 2I). Furthermore, ghrelin-treatment significantly down-regulated Interleukin 4 (Il4) at 4 days (p = 0.0097) and 20 days (p = 0.0215) post-surgery (Fig 2C). At 4 days post-surgery, interleukin-13 receptor alpha-2 (Il13ra2, p = 0.0481) mRNA levels were significantly decreased in ghrelin-treatment samples compared to the controls (Fig 2H). In contrast, Smad3 (p = 0.0023), Smad6 (p = 0.0093) and Smad7 (p = 0.0010) were significantly up-regulated in the ghrelin-treated tissues (Fig 2D, 2E and 2F). Expression of intercellular adhesion molecule 1 was significantly down-regulated at 4 days (Icam-1, p = 0.0215) and 20 days (Icam-1, p = 0.0120) post-surgery in ghrelin-treated samples in comparison to the controls (Fig 3A). No significant changes in vascular cell adhesion protein 1 (Vcam-1) gene expression were observed between ghrelin and saline-treated samples (Fig 3B). Ghrelin-treatment significantly down-regulated Mapk14 (p38α) at 20 days post-surgery (Mapk14, p = 0.0399) and Mapk14 mRNA levels were also reduced in ghrelin treated samples compared to the controls at 1 day post-surgery (Mapk14, p = 0.0579) (Fig 4A). Student t-test analysis showed that Smad4 (p = 0.0676) and Myc (p = 0.0435) mRNA levels were also reduced in ghrelin-treated samples compared to the controls at 1 day post-surgery (S1 Fig). In addition, tumor necrosis factor (Tnf, p = 0.0602) mRNA levels were decreased in ghrelin treated samples at 4 days post-surgery (S1 Fig).

### Western blot analysis showed altered protein expression in ghrelin-treated ischemic buttons

Immunoblotting was used to validate the gene expression data analysis. Since phosphorylation is a post-translational modification that regulates the activity of MAPKs, we examined the effect of ghrelin on phospho-p38 MAPK. The ischemic buttons showed increased protein levels of phospho-p38 MAPK, but this increase was attenuated by treatment with ghrelin (phospho-p38 MAPK, p = 0.0526) (Fig 4B and 4C).

**Fig 5:**
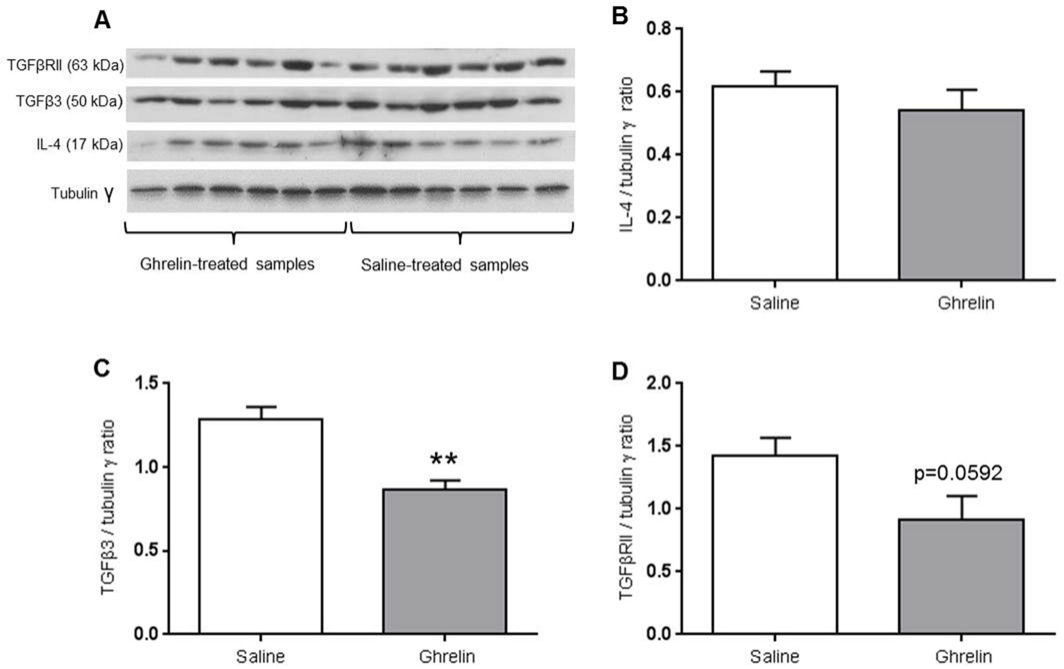
Western blot analysis of TGFβ3, TGFβR2 and IL-4 protein levels. The protein levels of TGFβ3 (A,C) and TGFβR2 (A,D) were reduced in the ghrelin-treated samples (n = 6) compared to the saline-treated samples (n = 6) at 4 days post-surgery. No significant differences were detected for IL-4 protein levels (A,B). Data are expressed as mean ± SEM and analyzed by Student’s t-test (** p <0.01).

TGFβ3 (p = 0.0013) and TGFβR2 (p = 0.0592) protein levels were decreased in ghrelin-treated ischemic buttons compared to controls at 4 days post-surgery (Fig 5A, 5C and 5D).

Phospho-SMAD2 and phospho-SMAD3 are the activated form of SMAD2 and SMAD3. The total SMAD2 and SMAD3 protein levels remained unchanged by ghrelin treatment. Phospho-SMAD3 (p = 0.0978) protein levels were decreased in ghrelin-treated samples compared to the control at 4 days post-surgery (S2 Fig).

No significant differences were detected in IL-4 (Fig 5A and 5B), phospho-SMAD2 (S2 Fig), ICAM-1 and VCAM-1 protein levels between ghrelin and saline-treated groups (data not shown).

### Pathway and network analysis defined by IPA revealed inhibition of TGF-β signaling pathways in ischemic buttons after chronic ghrelin administration

**Fig 6:**
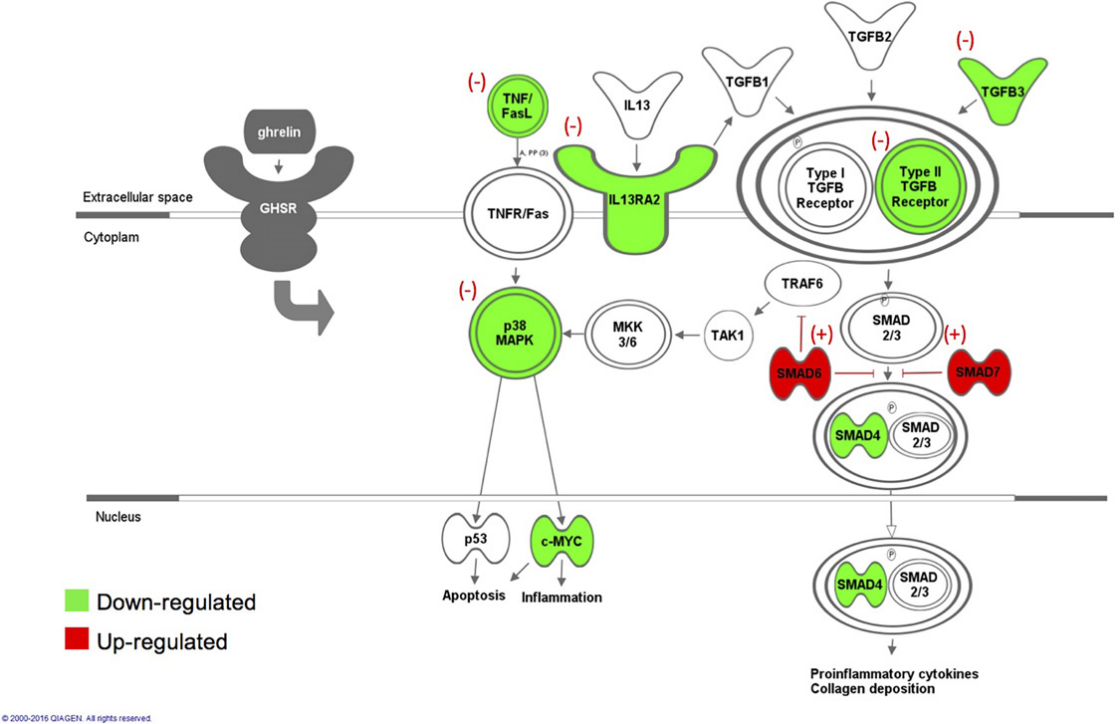
Ghrelin-induced anti-inflammatory effect is mediated by inhibition of TGF-β/Smads and p38-MAPK signaling pathways. A representative diagram characterizing the possible pathways by which ghrelin modulates the inflammatory response. Ghrelin binding to the GHSR receptor negatively modulates TGF-βR2, TGF-β3, TNF and IL13RA2 and up-regulates the inhibitory proteins SMAD7 and SMAD6, establishing the inhibition of TGF-β/Smads and p38-MAPK signaling pathways. This diagram was designed loading the fold change of ghrelin-treated samples versus controls into the Ingenuity pathway analysis software. Red highlighted genes represent overexpression and green represents underexpression of the factors of these pathways after ghrelin administration.

The p-value significant up- or down-regulated genes were subsequently subjected to Ingenuity Pathway Analysis (IPA) to obtain profiles of altered signaling pathways and to identify gene interaction networks. IPA revealed that ghrelin administration promotes inhibition of TGF-β/Smads and p38-MAPK signaling pathways (Fig 6).

## Discussion

Bianchi E et al, 2015 established a new mouse surgical model for the induction of adhesions that is consistent and easily reproducible. This new surgical model showed that ghrelin administered intraperitoneally prevents post-operative adhesion formation in a GHSR-1a receptor-dependent manner [25]. In the present study, this surgical model was employed to define altered molecular events regulating post-operative adhesion formation after intraperitoneal administration of ghrelin. The genesis of adhesion formation is due to a trauma/injury that results in the initiation of an inflammatory response leading to the formation of a fibrin matrix [5]. During normal peritoneal healing, the fibrin bands are broken down by fibrinolysis into fibrin degradation products; alternatively, during abnormal healing, ischemia results in a reduction of fibrinolytic activity leading to adhesion formation [5].

Previous findings elucidated the importance of the peritoneal fibrinolytic system in the regulation of adhesion formation. Down-regulation of fibrinolytic activity results in increased adhesions, while up-regulation results in a reduction of adhesions [27–29]. Plat mRNA expression was significantly reduced in ghrelin-treated samples at 1 day, resulting in a down-regulation of the fibrinolytic activity. Thus, the mechanism by which ghrelin decreases adhesion formation is not associated with the activation of the fibrinolytic pathway. Additionally, significant upregulation of the matrix metalloproteinases mRNA levels in ghrelin-treated samples compared to the controls was not detected (S1 Fig).

Since alterations in fibrinolysis did not explain the ghrelin-induced amelioration of adhesions, the role of inflammation was investigated by measuring the mRNA expression levels of several interleukins and cytokines that are implicated in the initial phase of the inflammatory process. The ability of ghrelin in alleviating post-operative adhesiogenesis may be associated with effects on these initiators of inflammation and a resulting down-regulation of Tgfb3 mRNA and protein levels (Fig 6). Expression of all three TGF-β isoforms increase after peritoneal injury at different times. TGF-β3 protein levels were minimally expressed after surgery increasing with the formation of adhesions. Exogenous addition of TGF-β3 at the time of surgery significantly increased adhesion formation in the mouse [13].

Our findings showed that TGFbr2 mRNA and protein levels were also decreased in ghrelin-treated peritoneal ischemic tissue compared to the controls. This ghrelin-induced decreased expression of TGF-βR2 and TGF-β3 may attenuate TGF-βR1 pathway activation, resulting in the inhibition of TGF-β activity. Canonical and non-canonical TGF-β pathways are involved in the activation of myofibroblasts and extracellular matrix synthesis and deposition [30]. TGF-βR2 is required for intracellular signal transduction through the formation of a heterotetrameric complex with TGFβ-R1 (Fig 6). The TGFβ-R2 receptor is a constitutively active kinase whereas the TGF-βR1 needs to be activated by the TGFβ-R2. After ligand-induced formation of the heteromeric complex, TGFβ-R2 phosphorylates TGF-βR1 at glycine and serine/threonine residues. This phosphorylation event changes the conformation of the TGF-βR1 receptor, thereby activating its kinase. The activated TGF-βR1 receptor propagates the intracellular signal by phosphorylating specific proteins (SMADs) (Fig 6) [31].

Studies in both humans and animal models are supportive of these findings, in which the overproduction of TGF-β was correlated with pathological tissue fibrosis and adhesion formation [13]. Similarly, inhibition of TGF-β activity reduces adhesion formation [13, 32]. The ghrelin-induced blockage of TGF-β activity was also confirmed by up-regulation of Smad7 at 4 days post-surgery in our surgical mouse model. Smad7 is an inhibitor of TGF-β signaling by preventing Smad3 and/or Smad2 phosphorylation and recruitment of Smad2/Smad3 complex (Fig 6). Smad7 protects against TGF-β induced fibrosis in several organs [33–35]. Ghrelin-induced up-regulation of Smad3 gene expression at 4 days post-surgery may be a compensatory effect generated by the inhibitory effect of Smad6 and Smad7 on TGF-β signaling. To examine the TGF-β downstream signaling molecules mediating the effect of ghrelin, western blot analysis was conducted to quantify the phospho-SMAD2 and phospho-SMAD3 protein levels on the total levels of SMAD2 and SMAD3 at 4 days post-surgery. In comparison to the control samples, ghrelin-treated samples were suggestive of an attenuation of phospho-SMAD3 protein levels relative to the total SMAD3 proteins levels at 4 days post-surgery (S2 Fig).

TGF-β may also work through an alternative non-Smad signaling pathway to convey the same invasive fibrinogenic signals [36]. Tumor necrosis factor (TNF)-receptor-associated factor 6 (TRAF6) and TGF-β associated kinase I (TAK1) are crucial for the activation of MAPK signaling [37].

The current study revealed that ghrelin administration significantly decreases Tnf, Mapk14 (p38α) and Myc mRNA levels and increases Smad6 mRNA levels. These results suggest that the ghrelin anti-adhesion effect is also mediated by down-regulation of p38-MAPK signaling pathway (Fig 6). c-Myc is a downstream target of p38α and it is known to be involved in inflammation [38] and apoptosis [39]. p38-MAPK signaling is a compensatory pathway that amplifies the response to TGF-β via a non-canonical pathway in case of reduced expression of TGF-β receptors [40]. Jung S.M. et al. 2013, showed that Smad6 negatively regulates the activation of the non-canonical TGF-β pathway through recruitment of the de-ubiquitinase A20 to TRAF6 [41]. The inhibition of p38-MAPK results in a reduction of alpha-SMA expression which is a key factor involved in the differentiation of fibroblasts to a contractile myofibroblastic phenotype [42].

Il-4 mRNA expression was significantly decreased at 4 and 20 days post-surgery in ghrelin-treated peritoneal ischemic buttons compared to controls. IL-4-activated M2 macrophages are involved in the peritoneal fibrotic process with peritoneal M2 macrophages acting as potential targets for the interventional therapy of peritoneal fibrosis. The IL-4 cytokine promotes alternative activation of macrophages into the M2 phenotype and inhibits classical activation of macrophages into the M1 phenotype. An increase in M2 macrophages, also known as repair macrophages, is coupled with the secretion of TGF-β. The release of arginase, proline, polyaminases and TGF-β by the activated M2 cells is associated with peritoneal fibrosis [43].

In addition, IL13RA2 mRNA was significantly decreased at 4 days post-surgery in ghrelin-treated peritoneal ischemic buttons compared to controls, suggesting that the anti-fibrogenic response of ghrelin is potentiated by down-regulation of IL13RA2 signaling upstream. IL-13 induces monocytes and macrophages to produce TGF-β1, a potent regulator of extracellular matrix deposition and tissue remodeling [44]. Fichtner-Feigl S et al. 2006, suggested that Il13RA2 signaling is an important therapeutic target for the prevention of fibrosis [45].

Adhesion formation is associated with an increase of local inflammatory mediators, including ICAM-1 and VCAM-1 [46]. The present study shows that ghrelin significantly decreases Icam-1 mRNA levels in peritoneal ischemic tissue at 4 and 20 days post-surgery. The first step in adhesion formation is the initiation of a local inflammatory response at the site of injury via expression of ICAM-1 and VCAM-1 by mesothelial cells lining the peritoneal cavity to facilitate leukocyte migration through the wall of the blood vessel [5].

In conclusion, these experiments suggest that ghrelin inhibits the TGF-β/Smads and p-38 MAPK signaling pathways activated during the inflammatory response at the onset of injury before the granulation-remodeling phase occurs. We provide evidence that ghrelin reduces up-stream collagen deposition and myofibroblastic differentiation via down-regulation of pro-inflammatory factors, TGFβ3, TGFβR2 and up-regulation of inhibitor proteins such as Smad7 and Smad6. Therefore, the attenuation of extracellular matrix component production and differentiation of fibroblasts into myofibroblasts via inhibition of the inflammatory process at the onset of injury rather than up-regulation of fibrinolytic activity may be a viable therapeutic strategy for the prevention of post-surgical adhesions.

## Supporting Information

S1 Fig Gene expression of peritoneal ischemic tissues exposed to ghrelin for 1, 4 and 20 days. Data are presented as fold change of ghrelin-treated samples on controls over the geometric mean of all the Housekeeping genes (Actb, B2m, Gapdh, Gusb and Hsp90ab1). Data are analyzed using the ΔΔCt method and expressed as mean ± SEM. Student’s t-test (* p <0.05).
(TIF)

S2 Fig Effect of ghrelin on TGF-β/Smads downstream signaling pathway at 4 days post-surgery. (A, B) Immunoblotting of phospho-SMAD2 and phospho-SMAD3 in ghrelin and saline-treated peritoneal ischemic buttons. (C) Relative phospho-SMAD2 over total SMAD2 and (D) phospho-SMAD3 over total SMAD3 protein levels. (D) The phospho-SMAD3/SMAD3 protein level ratio was reduced in ghrelin-treated samples (n = 5) compared to the controls (n = 5) at 4 days post-surgery. (C) No significant differences were detected for phospho-SMAD2/SMAD2 protein level ratio (A,B). Data are expressed as mean ± SEM and analyzed by Student’s t-test.
(TIF)
