# Supplementary figures and images for: Ghrelin Inhibits Post-Operative Adhesions via Blockage of the TGF-β Signaling Pathway
Source: PLoS One. 2016 Apr 15;11(4):e0153968. doi: 10.1371/journal.pone.0153968 (PMC4833425; doi:10.1371/journal.pone.0153968)

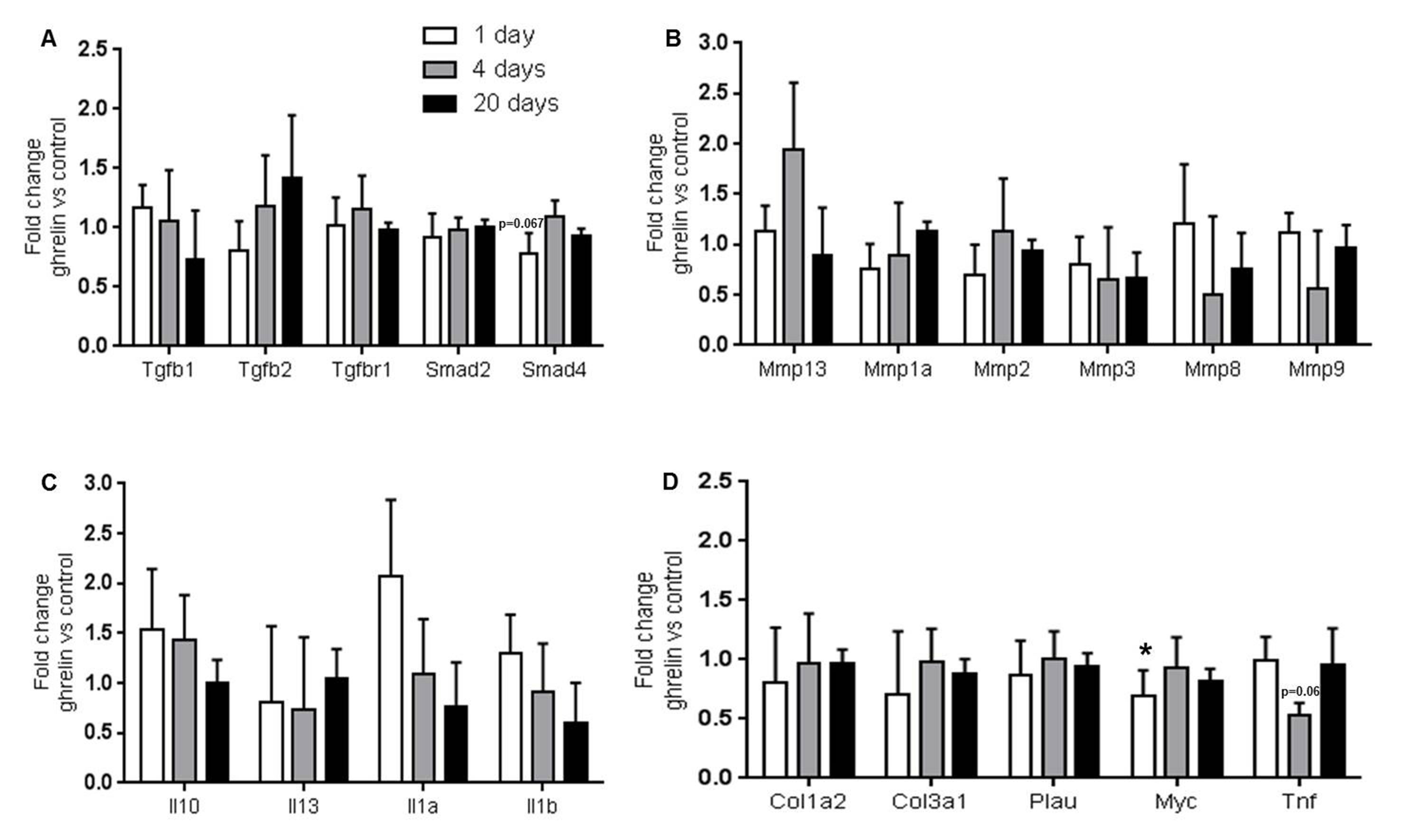

Supplement: S1 Fig — Data are presented as fold change of ghrelin-treated samples on controls over the geometric mean of all the Housekeeping genes (Actb, B2m, Gapdh, Gusb and Hsp90ab1). Data are analyzed using the ΔΔCt method and expressed as mean ± SEM. Student’s t-test (* p <0.05). (TIF) [file pone.0153968.s001.tif]

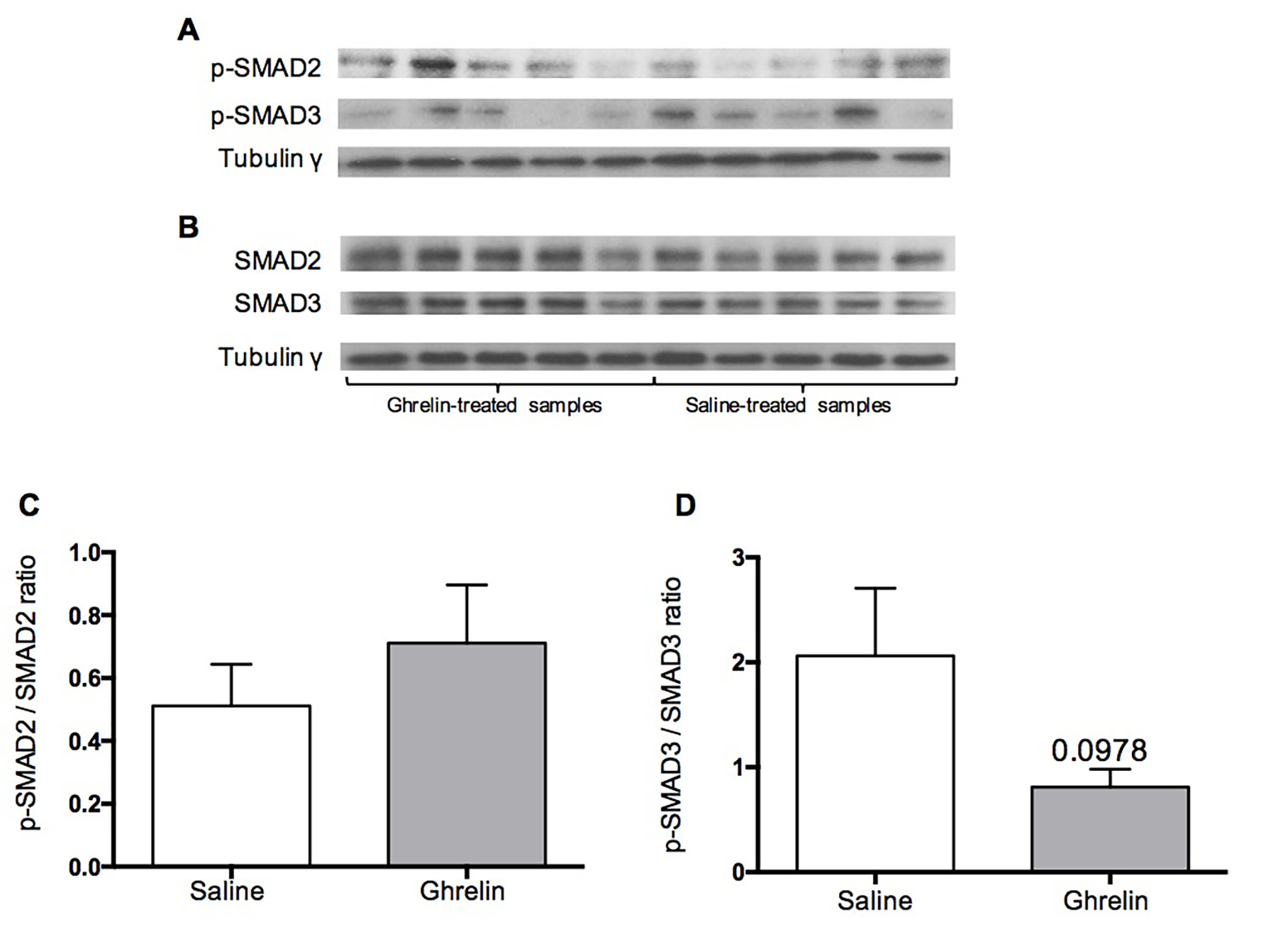

Supplement: S2 Fig — (A, B) Immunoblotting of phospho-SMAD2 and phospho-SMAD3 in ghrelin and saline-treated peritoneal ischemic buttons. (C) Relative phospho-SMAD2 over total SMAD2 and (D) phospho-SMAD3 over total SMAD3 protein levels. (D) The phospho-SMAD3/SMAD3 protein level ratio was reduced in ghrelin-treated samples (n = 5) compared to the controls (n = 5) at 4 days post-surgery. (C) No significant differences were detected for phospho-SMAD2/SMAD2 protein level ratio (A,B). Data are expressed as mean ± SEM and analyzed by Student’s t-test. (TIF) [file pone.0153968.s002.tif]
